# Influence of Twist Channel Angular Pressing Process on Microhardness and Microstructural Behavior of Explosively Welded Al/Cu Plates

**DOI:** 10.3390/ma19020302

**Published:** 2026-01-12

**Authors:** Krzysztof Żaba, Łukasz Kuczek, Ilona Różycka, Ondřej Hilšer, Tomasz Trzepieciński, Kinga Ortyl

**Affiliations:** 1 Department of Metal Working and Physical Metallurgy of Non-Ferrous Metals, Faculty of Non-Ferrous Metals, AGH University of Krakow, al. Adama Mickiewicza 30, 30-059 Cracow, Poland; lukasz.kuczek@agh.edu.pl (Ł.K.); k.ortyl2510@gmail.com (K.O.); 2 Department of Materials Science and Engineering of Non-Ferrous Metals, Faculty of Non-Ferrous Metals, AGH University of Krakow, al. Adama Mickiewicza 30, 30-059 Cracow, Poland; rozycka@agh.edu.pl; 3 Faculty of Mechanical Engineering, VSB-Technical University of Ostrava, 17. listopadu 2172/15, 708 00 Ostrava, Czech Republic; ondrej.hilser@vsb.cz; 4 Department of Manufacturing Processes and Production Engineering, Faculty of Mechanical Engineering and Aeronautics, Rzeszów University of Technology, al. Powst. Warszawy 8, 35-029 Rzeszów, Poland; tomtrz@prz.edu.pl

**Keywords:** explosive welding, microhardness, microstructure, severe plastic deformation, twist channel angular pressing, Al/Cu plates

## Abstract

Due to their unique properties resulting from the combination of metals with different properties, bimetallic sheets are desirable in the energy, petrochemical, and shipbuilding industries. In this article, explosively welded EN AW-1050/Cu-ETP (Al/Cu) plates were used as the test material. One of the greatest advantages of Al/Cu bimetallic plates is their high deformability, which allows for easy plastic forming. The aim of this study was to determine the effect of severe plastic deformation on the microstructure and microhardness of explosively welded EN AW-1050/Cu-ETP plates. Bimetallic samples were processed using the Twist Channel Angular Pressing (TCAP) process. This process consisted of varying the number of passes and the sample orientation relative to the helical exit channel of the TCAP die. For comparative purposes, a microstructural analysis and the microhardness testing of the as-welded samples were also carried out. Microstructural analysis of TCAP-processed samples showed that the sample deformed along route Bc exhibited the most deformed weld interface profile. No cracking or delamination was observed in the Al/Cu interfacial transition layer of TCAP-processed samples. The number of passes and orientation of the bimetallic material relative to the die exit channel affected the final microhardness in the individual layers of explosively welded EN AW-1050/Cu-ETP bimetallic plate.

## 1. Introduction

The demand for bimetal plates continues to grow due to their unique properties. Bimetal sheets are widely used in the power, electronics, chemical, petrochemical, and shipbuilding industries. Al/Cu bimetal sheets are characterized by high strength and corrosion resistance, making them widely used in the production of machine components in the petrochemical and power industries [1,2]. They also exhibit high thermal and electrical conductivity, allowing for effective heat dissipation and minimizing energy losses in the energy industry. Cu-based clad conductors are very efficient for applications using the alternating current, due to the occurrence of the “skin effect” [3]. Multi-layer Al/Cu sheets are highly susceptible to plastic forming. They are resistant to high temperatures and exhibit good weldability [4,5]. Roostaei and Darabi [6] reported that explosively welded Al/Cu/Al sheets exhibit better formability than constitutive layers. The improvement in formability is related to the strong binding between layers. The combination of high electrical conductivity of copper and low mass of aluminum is desirable in the power industry for components like heat exchangers, busbars, and electric connectors [7]. However, pure metals are characterized by relatively low strength properties, which can hinder their use as conductors, where, in addition to good electrical conductivity, good tensile strength is also required. In the context of conductive materials, HSHC (High Strength High Conductivity) materials are gaining increasing interest, particularly copper. These materials, while possessing good conductivity of 50–95% IACS (International Annealed Cu Standard), are also characterized by high strength properties—from 1.5 to even 4 times the strength of pure copper.

Bimetallic sheets are fabricated using methods such as hot and cold rolling, explosion welding, friction welding, diffusion bonding, hot-wire gas tungsten arc welding (GTAW), and hot pressure welding, which create a metallurgical bond between two different metals [8,9,10,11]. In conventional welding or rolling methods, differences in melting points and thermal expansion cause cracks, oxidation, or discontinuities in microstructure. Explosive welding allows for the effective joining of metals with different physical and chemical properties [12]. This process occurs without melting the joined materials and without the occurrence of a heat-affected zone [13]. Additionally, the shock wave creates characteristic wave-like interface that increases the contact surface and resistance to separation [14,15]. In the near-interface area, both the microstructure and properties of the material change [16,17]. Grains can elongate, and with sufficiently high kinetic energy, this can be converted into thermal energy and formation of local melting areas. As a result, intermetallic compounds (IMCs) are formed in these locations. Bakhtiari et al. [18] reported that some interface compounds were separated from the aluminum layer by a thin copper-rich region, which was the result of jetting formation and entrapment between the waviness of the interface combined with a local increase in adiabatic temperature. Explosive welding technology, as the most universal method for producing various types of intermetallic joints, offers the possibility of obtaining material combinations impossible to obtain by other methods [19,20]. The versatility of this method results from the fact that it is possible to combine metals with significantly different properties such as melting point, density, and chemical activity [21].

Improving the properties of metallic materials, including bimetallic plates, can be achieved through severe plastic deformation (SPD), which is primarily used to significantly reduce the grain size in the material, thereby improving its mechanical properties without changing its chemical composition [22,23]. The phenomenon of strain hardening should also be mentioned, which is associated with an increase in dislocation density in a material subjected to SPD. According to the Hall–Petch effect, the smaller the grains, the larger the grain boundary and the higher the tensile strength and hardness [24]. SPD methods include equal channel angular pressing (ECAP), twist chennel angular pressing (TCAP), parallel tubular channel angular pressing (PTCAP), cyclic extrusion compression (CEC), high pressure torsion (HPT), accumulative roll bonding (ARB), multi-directional forging (MDF), constrained extrusion/channel extrusion, twist extrusion, and many others [25,26,27]. Among these methods, TCAP is a modification of the ECAP method using an angular exit channel [28]. This channel is twisted (spiral), which induces additional high shear strain along the entire cross-section of a workpiece during the TCAP process [29]. In TCAP, the twisted channel introduces continuous shear along the entire length of the sample, so deformation is more uniform throughout the material volume than that in ECAP. The combination of angular and torsional strain increases the overall deformation intensity, resulting in smaller grains than in the ECAP method after the same number of passes. Various processing routes are used in the TCAP process: route A (the batch is not rotated), route B_A_ (batch is rotated by 90° in an alternate orientation), route B_C_ (batch is rotated by 90° in the same orientation), and route C (the batch is rotated by 180° after each pass) [23].

In the literature, there are few approaches to SPD of Al/Cu bimetal workpieces. Shaeri et al. [30] observed that the structural homogeneity and strength of EN AW-7075 aluminum alloy increased with increasing the copper tube casing thickness. Varadala et al. [31] investigated the deformation behavior of EN AW-5083 aluminum alloy circular bills of with and without Cu casing. These authors reported that the use of the Cu casing reduces the amount of deformation loads. Ebrahim et al. [32] investigated deformation behavior of EN AW-6082/Cu bimetallic tubes in shear punch test. After three passes of parallel tube-shaped channel angular pressing, the shear strength between the EN AW-1050/Cu composite layers was increased by 208% [33]. The SPD method allowed us to obtain sound bonding between the tubes. Shirzad et al. [34] developed the parallel tubular channel angular pressing process to fabricate an EN AW-1050/Cu bimetallic tubes. Ultrasonic vibrations of the punch allowed for a reduction in the forming force. Ghadimi et al. [35] examined the properties of EN AW-6061/Cu bimetallic tubes produced by ECAP. These authors reported that the hardness of the ECAP-processed material increased more than 120%. Żaba et al. [36] investigated the effect of the roll bonding process on the mechanical properties of EN AW-1050/M1E copper sheets. It was found that the orientation of the strip samples with respect to the sheet rolling direction determines the stiffness of bimetallic sheets. Kocich [3,37] studied the effect of the TCAP process on the microstructure and selected properties of Al/Cu composites in the form of aluminum billets reinforced by Cu wires. It was found that the multiple-pass TCAP processing increased the quality of bonding of both the component metals. Parimi et al. [38] investigated the deformation characteristics of Al/Cu billets in a multiple channel-die compression process. This process allowed the creation of nano-structured bulk copper material. Mirzakouchakshirazi et al. [39] investigated shear bond strength of Al/Cu bimetallic rods using the ECAP process. As a result of ECAP processing, the shear bond strength between copper and aluminum increased by approximately 150%.

The main goal of this study was to determine the effect of the TCAP process on the microstructure, particularly whether any discontinuities occurred that could contribute to later delamination of the material, and microhardness of explosively welded EN AW-1050/Cu-ETP plates. The morphology of the Al/Cu interfacial layers of TCAP-processed samples was examined via a scanning electron microscope (SEM). The change in the mechanical properties of the sheets resulting from the TCAP process was assessed based on a grid of microhardness measurements on the longitudinal and transverse sections of the samples.

## 2. Materials and Methods

### 2.1. Test Material

The test material Cu-ETP copper (base material) and EN AW-1050 aluminum alloy (clad) were explosively welded with a thickness of 10 mm. The base materials were 5 mm thick. The explosive welding process was carried out under industrial conditions at Explomet Gałka, Szulc Sp.k. (Opole, Poland). The requirements of the EN 573-1 standard [40] regarding the chemical composition of the EN AW-1050 aluminum alloy plate (flyer plate) are presented in Table 1. Table 2 contains the requirements for the chemical composition of the Cu-ETP copper plate (base metal) material according to the EN 1652 standard [41]. The test material is characterized by high thermal and electrical conductivity, which allows for efficient heat dissipation and minimizes energy losses in the power industry.

Samples for TCAP testing were cut from explosively welded bimetallic plates. The sample dimensions were 10 mm (width) × 10 mm (height) × 50 mm (length). To fit the TCAP channel dimensions (15 mm × 15 mm), the cut samples were inserted into 2.5 mm thick shells made of 1050A aluminum alloy in the annealed condition. 

### 2.2. Experimental Procedures

#### 2.2.1. Microstructure Characterization

A SU70 (Hitachi, Tokyo, Japan) scanning electron microscope was used for microstructural examination of as-welded and TCAP-processed samples. The microstructure of the samples was examined in two perpendicular planes relative to the Al/Cu interface. The samples were cut transversely and longitudinally to the Al/Cu interface. Metallographic sections were prepared by (i) grinding with 200 to 4000 mm grit paper, (ii) polishing with DiaDuo diamond paste, and (iii) polishing in an oxide polishing slurry. Energy-Dispersive X-Ray Spectroscopy (EDS) was used to detect the chemical composition on the surface of the metallographic sections and in selected spots of the samples.

#### 2.2.2. Vickers Microhardness

**Figure 1:**
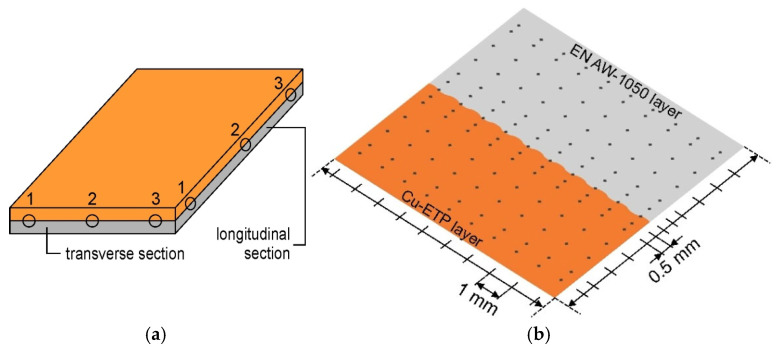
(**a**) Microhardness measurement locations and (**b**) microhardness measurement grid.

Vickers microhardness measurements were performed at three locations on the longitudinal and transverse sections of as-received and TCAP-processed samples. The measurement was performed in two directions due to the possibility of anisotropy in the mechanical properties of the material resulting from the nature of the explosive welding of aluminum and copper plates. In this type of process, both the movement of the flyer material (associated with the explosion front), jetting, and plastic deformation are highly directional, which can affect the product’s properties. Samples are designated XY, where X denotes the location from which the sample was cut (Figure 1a) and Y denotes the cutting orientation (longitudinal—L and transverse—T, to the joint). Vickers microhardness measurements were performed using a Tukon 2500 hardness tester at a load of 4.98 N. A grid of measurements was performed on the surface of each sample (Figure 1b). Measurements were taken near the Al/Cu interface and at the outer surfaces of the bimetallic samples with a resolution of 0.5 mm, while inside the base materials (Al and Cu), the measurement was taken with a resolution of 1 mm. In the direction parallel to the interface, the measurement was performed with a constant step of 1 mm (Figure 1b).

#### 2.2.3. Twist Channel Angular Pressing

Twist Channel Angular Pressing was performed on a LabTest 5.2000CT (Labortech, Opava, Czech Republic) press with a maximum load of 2 MN, located at the VŠB—Technical University of Ostrava (Faculty of Mechanical Technology). The tests were performed with varying sample orientations relative to the die entry channel. TCAP samples were cut transversely to the Al/Cu interfacial transition layer. In the multi-pass process, the sample was trimmed and ground before each subsequent pass. Table 3 presents the sample designations and the corresponding TCAP process parameters. This designation allowed for the evaluation of the effect of the number of passes and TCAP route (TCAP1 and TCAP2), and the orientation of the bimetallic material (TCAP1, TCAP3, and TCAP4) on the microstructure and microhardness of the TCAP-processed samples.

**Figure 2:**
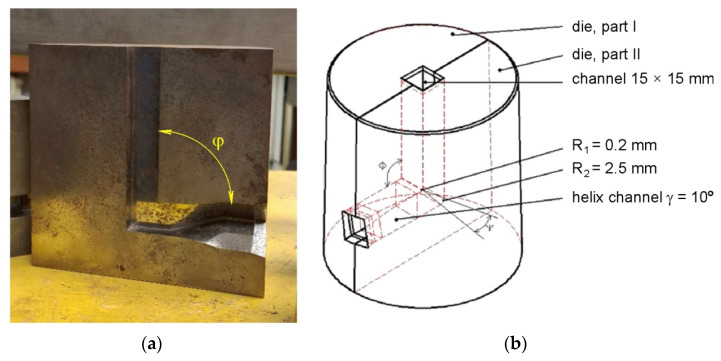
(**a**) Photograph of the TCAP die (φ = 90°) and (**b**) die geometry.

TCAP experiments were conducted at ambient temperature using a die with a channel angle of φ = 90° (Figure 2a) and a twist angle of γ = 10° (Figure 2b). 

**Figure 3:**
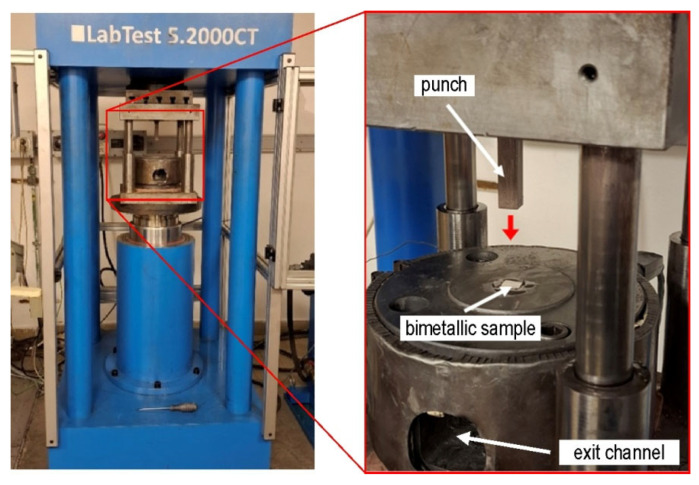
Setup of TCAP process.

**Figure 4:**
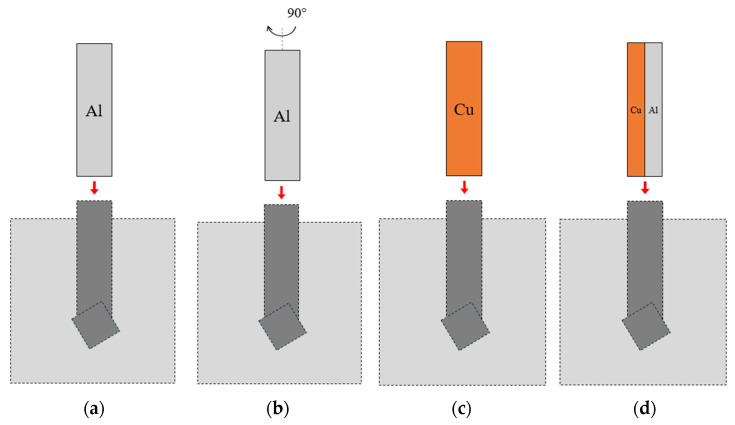
Sample orientation during TCAP processing: (**a**) TCAP1, (**b**) TCAP2, (**c**) TCAP3, and (**d**) TCAP4.

The ram speed was 40 mm/min. To reduce the coefficient of friction between the sample and the die, graphite grease was used. The samples were placed sequentially in the die entry channel (Figure 3), and then the press was engaged. The lowering punch forced the material through the twist channel in the die. The first sample, TCAP1 (Figure 4a), was placed in the entry channel with the Al layer facing the die exit opening. The second sample, TCAP2 (Figure 4b), was processed in the same manner, but to increase strain accumulation, the sample was formed twice. The second pass was made according to route Bc. The third sample (TCAP3) and fourth sample (TCAP4) were placed in the entry channel of the die with the Cu and the Al/Cu interface facing the exit channel of the die (Figure 4c,d).

## 3. Results and Discussion

### 3.1. Microstructure of As-Welded Sample Material

**Figure 5:**
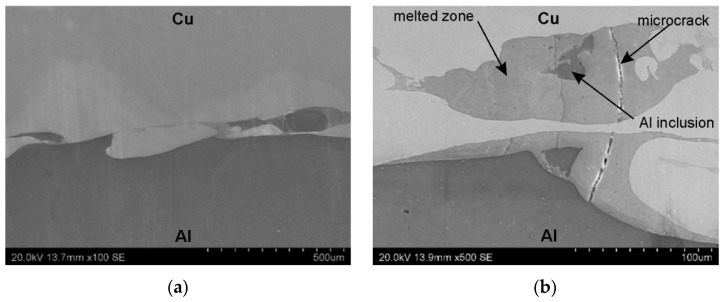
The interface layer formation in transverse section of the explosively welded plates at different magnifications: (**a**) ×100 and (**b**) ×500.

**Figure 6:**
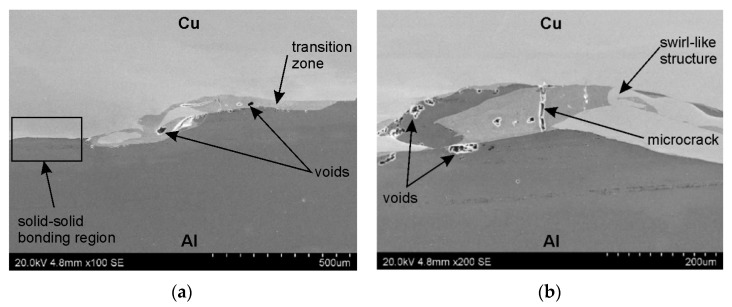
The interface layer formation in longitudinal section of the explosively welded plates at different magnifications: (**a**) ×100 and (**b**) ×200.

**Figure 7:**
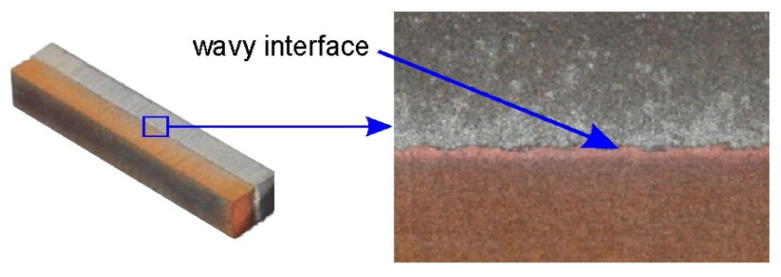
Morphology of the interface in explosively welded Al/Cu plates.

SEM micrographs showing the interface layer formation in transverse and longitudinal sections of the explosively welded plate are presented in Figure 5 and Figure 6, respectively. A wave-like interface resulting from explosion welding was observed in the joint zone (Figure 7). The wave-like interface in explosive welding is induced by shock wave instability. The wave-like morphology of the Al/Cu interface is typical for explosively welded bimetallic plates and is a result of Kelvin–Helmholtz instabilities [43] and the formation of a re-entrant jet [44]. Other mechanisms explaining the forming mechanism of the interface waves include the vortex shedding mechanism [45] and the interaction between the reflected compressive waves and rarefaction waves [46]. At higher magnifications, layers of aluminum plate material are visible in the transition layer. These layers migrated into the copper layer under the influence of the explosive shock wave and became trapped between the wavy layer of interface (Figure 5b and Figure 6b). Increase in temperature and local melting of components in the transition layer occurred due to severe dissipation of impact energy and severe friction conditions between the jet layer and contact surfaces. Therefore, the resulting cast structure was characterized by the presence of intermetallic compounds near the transition layer (Figure 6a,b). The explosive welding process involves joining metals with different chemical and physical properties. The detonation energy, which allows the materials to fuse, causes their permanent fusion, simultaneously creating transition zones and intermetallic phases. The source of pores in explosive welding is mainly trapped gas (air, and decomposition products of impurities) between the welded surfaces, which were not completely removed during the collision [21]. Porosity due to the Kirkendall effect arises from the uneven diffusion of vacancies at the interface between dissimilar metals, creating a porous zone at the interface. The presence of porosity may be a result of rapid growth of the intermetallic phases [14]. The participation of the gas during the wave formation led to the appearance of microcracks passing through the pores (Figure 5b) [47]. Although no standards related to the quality of the Al/Cu bimetallic materials connection were found, based on ASME standards for clad steel plates (e.g., ASME SA-263, ASME SA-264, ASME SA-265, and ASME SA-578/SA-578M), it can be concluded that the explosive welding procedure used is adequate. No significant differences were observed in the morphology of the Al/Cu interfacial transition layer in transverse and longitudinal sections.

**Figure 8:**
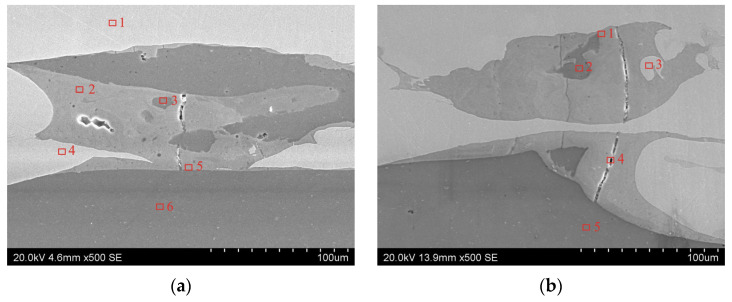
Areas for spot EDS analysis of explosively welded samples: (**a**) transverse section and (**b**) longitudinal section.

Areas for spot EDS analysis for the samples were located in transverse section (Figure 8a) and longitudinal section (Figure 8b) of explosively welded samples. The results of the spot EDS analysis of chemical composition in the area of Al/Cu interfacial layer of the explosively welded samples are presented in Table 4.

### 3.2. Microhardness of As-Welded Sample Material

**Figure 9:**
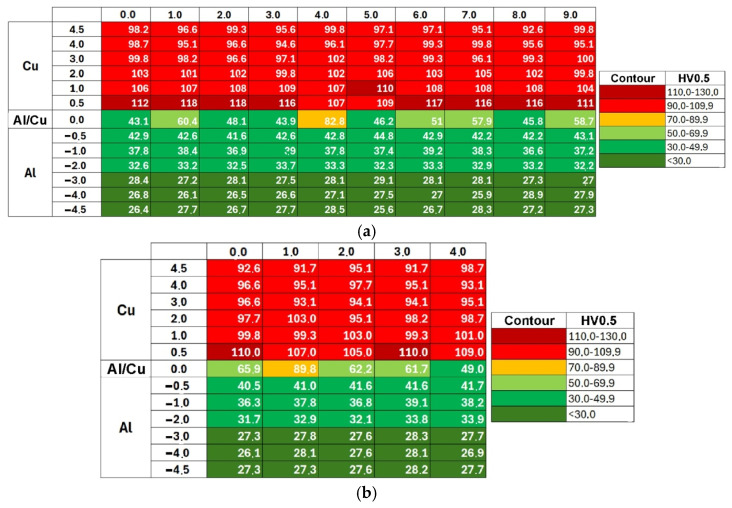
Vickers microhardness distribution map in the (**a**) 1L and (**b**) 1T samples.

Figure 9 shows the Vickers microhardness distribution map for samples 1L and 1T. Differences in measured values are presented using a contour plot, with dark green indicating the lowest microhardness and dark red the highest. The highest microhardness values were recorded in the Cu-ETP copper plate, near the Al/Cu interfacial transition layer. Copper is characterized by greater work hardening under cold deformation conditions than EN AW-1050 aluminum alloy [48]. The further from the Al/Cu interface, the lower the microhardness of both materials. Although the presented microhardness map (Figure 9) refers to samples 1L and 1T, similar conclusions can be observed for the remaining samples.

**Figure 10:**
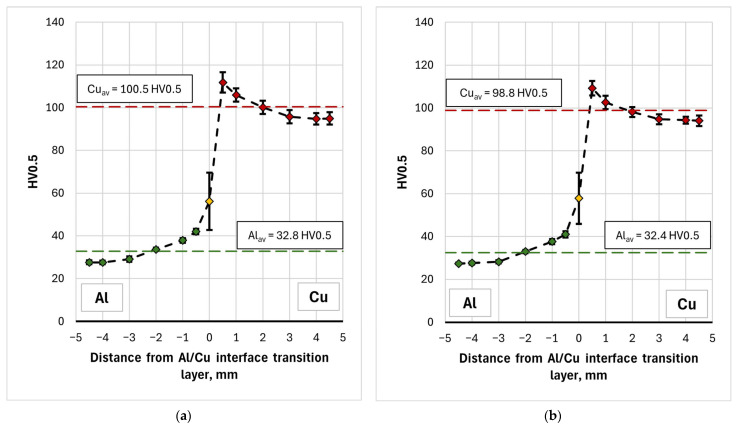
Microhardness distribution on the (**a**) longitudinal and (**b**) tranverse section of the as-welded samples.

The microhardness measurement results for samples 1L-3L and 1T-3T are also presented as graphs of the mean value (in a line parallel to the interface) versus distance from the Al/Cu interfacial transition layer (Figure 10). The graphs clearly illustrate the increase in microhardness of the material near the Al/Cu interfacial transition layer, on both the EN AW-1050 plate and Cu-ETP plate sides. This can be related to the appearance of adiabatic shear bands (ASB), where local concentrated plastic deformation occurred [17] The presence of intermetallic compounds and allotting phases in the interface area were also found. The presence of the latter also significantly influences the appearance of a large range of hardness values in the tested layer, which is visible in the form of whiskers (defined as standard deviation) in the graphs. With distance from the Al/Cu interface, the microhardness decreases, reaching the value of the as-received material at the vicinity of outer surfaces of the bimetallic plate. It was also observed that measurements taken directly at the interface were characterized by the highest standard deviation (SD). Due to the wave-like character of the Al/Cu interfacial transition layer, when the indentation was made in a material containing a higher proportion of Cu-ETP material, the microhardness was overstated. When the indentation was made in a zone containing a higher proportion of EN AW-1050 plate material, the microhardness was understated. Moreover, the presence of intermetallic phases in the interface area could also have significantly influenced the variation of the measured microhardness in this area.

Hardness measurements of the material in the longitudinal (L) and transverse (T) directions revealed slight differences in the averaged HV0.5 value, particularly for the copper layer and Al/Cu interface. For Cu layer, the mean microhardness of the samples cut in the longitudinal (L) and transverse (T) directions was 100.5 HV0.5 and 98.8 HV0.5, respectively. For the interface area, they were equal to 56.1 HV0.5 and 57.8 HV0.5, respectively. Although in the case of copper layers the difference between the mean values was statistically significant (Welch’s *t*-test, *p* = 0.041), the effect size measure indicating how large the difference is between the two groups with respect to the variability of the results, was small (Cohen’s d = 0.251). Furthermore, the difference between the mean values was 1.6 HV0.5, with partially overlapping confidence intervals (CI) for the tested samples (CI_L_ = ± 1.1 HV0.5, CI_T_ = ± 1.3 HV0.5). In the case of the Al/Cu interface, statistical analysis showed no significant difference in microhardness values between the samples (*p* = 0.670, Cohen’s d = −0.131). This means that, from a practical point of view, the determined differences can be considered negligible and the tested material exhibit essentially isotropic properties in terms of its microhardness. 

### 3.3. TCAP-Processed Samples

#### 3.3.1. Morphology of Al/Cu Interface

**Figure 11:**
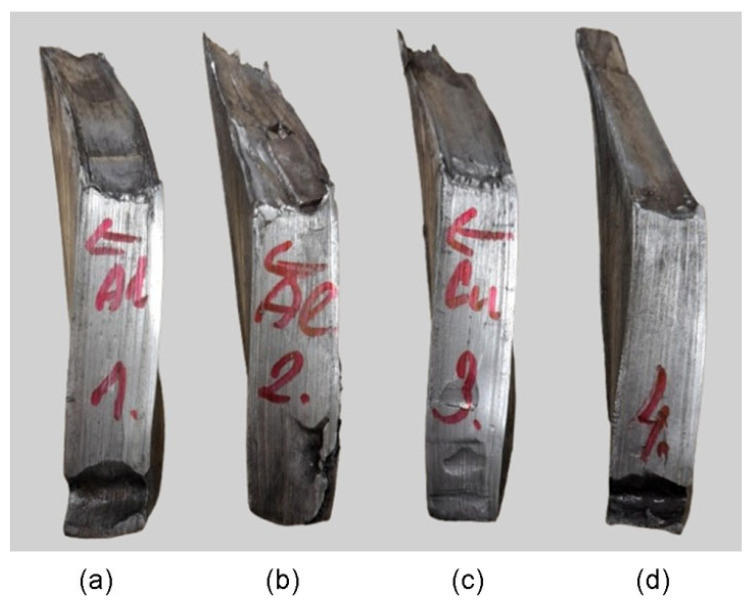
Shape of ECAP-processed samples: (**a**) TCAP1, (**b**) TCAP2, (**c**) TCAP3, and (**d**) TCAP4.

Due to the use of a TCAP die with a helix exit channel, the shape of the TCAP-processed samples was twisted (Figure 11). Four composite samples cut transversely to the Al/Cu interface were subjected to TCAP testing.

**Figure 12:**
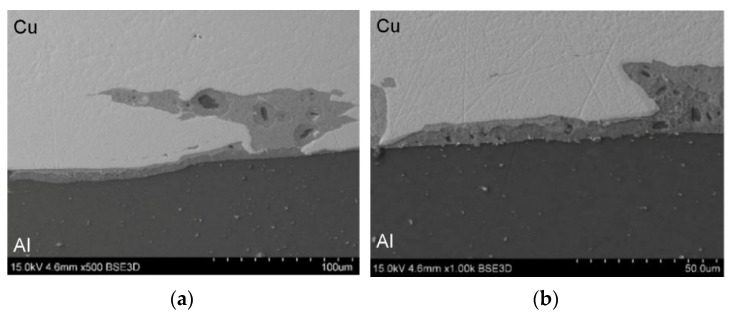
SEM micrographs of sample TCAP1 at different magnifications: (**a**) ×500 and (**b**) ×1000.

Figure 12 shows SEM micrographs of the Al/Cu interfacial transition layer of sample TCAP1. During TCAP process, the sample was oriented with the EN AW-1050 material layer facing the exit channel of the TCAP die (Figure 4a) and was deformed in a single pass. A few pores were observed in the vicinity of the Al/Cu interface, but the observed microstructure did not reveal any other significant differences from the as-welded material. The Cu-ETP material appears in the SEM micrographs as a light gray area, in contrast to the darker area of the EN AW-1050 material.

**Figure 13:**
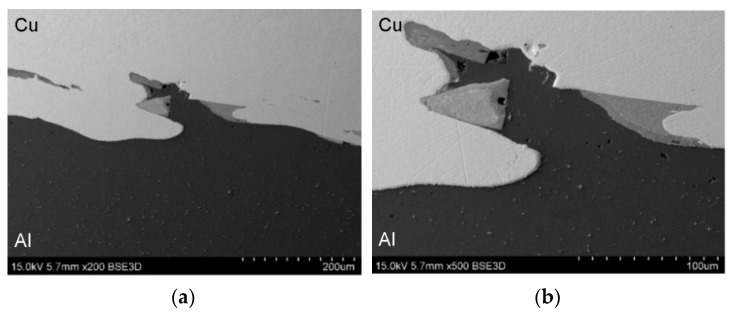
SEM micrographs of sample TCAP2 at different magnifications: (**a**) ×200 and (**b**) ×500.

Figure 13 presents the microstructure of the TCAP2 sample, which was deformed in two passes according to route Bc (Figure 4b). The microstructure showed isolated pores and a significantly more wavy and irregular character of the Al/Cu interfacial transition layer compared to the microstructure of the TCAP1 sample and the as-welded material. Despite the severe shear deformation realized in the two passes, no cracking or delamination was observed in the Al/Cu interfacial transition layer. This confirms the susceptibility of the tested bimetallic composite material to SPD without the risk of damage to the bond zone.

**Figure 14:**
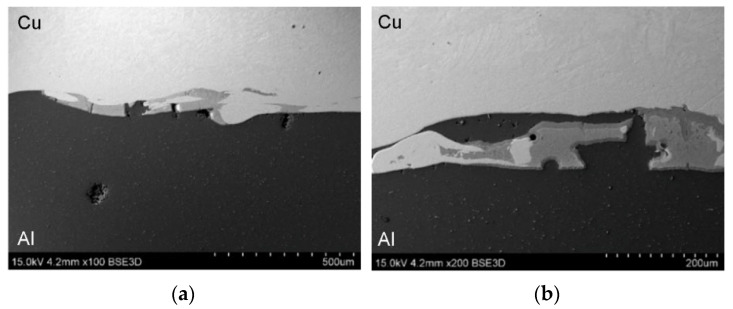
SEM micrographs of sample TCAP3 at different magnifications: (**a**) ×100 and (**b**) ×200.

**Figure 15:**
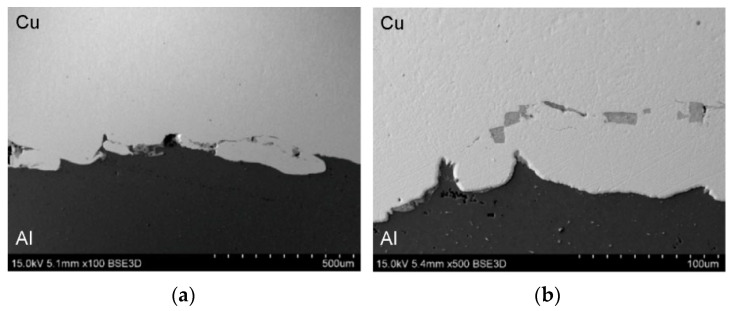
SEM micrographs of sample TCAP4 at different magnifications: (**a**) ×100 and (**b**) ×500.

During the TCAP process, the TCAP3 sample (Figure 14) was oriented with the Cu-ETP layer facing the exit channel of the die and was deformed in a single pass (Figure 4c). The TCAP4 sample (Figure 15) was oriented with the Cu-ETP material layer facing the exit channel of the die (Figure 4d) and was deformed in a single pass. The microstructure showed pores of similar size to those in the TCAP1 and TCAP2 samples. The Al/Cu transition layer boundary was characterized by a distinctly more irregular shape. It was also noted that the transition regions were not located directly adjacent to the Al/Cu interface, but inside the Cu-ETP material (Figure 15).

#### 3.3.2. Spot EDS Analysis

**Figure 16:**
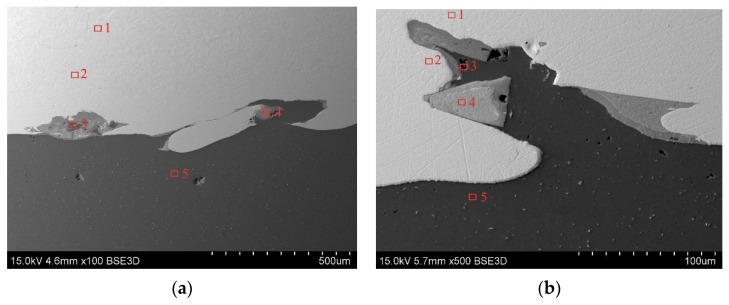

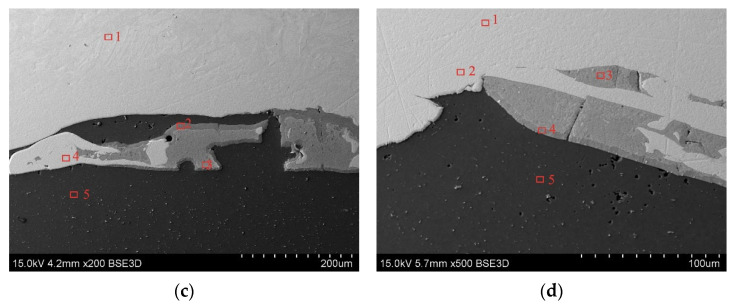
Areas for spot EDS analysis for the following samples: (**a**) TCAP1, (**b**) TCAP2, (**c**) TCAP3, and (**d**) TCAP4.

To precisely quantify the Cu and Al elemental content in the TCAP-processed samples, spot EDS analysis was performed. Measurements were taken at five points for each sample (Figure 16). Based on the results of spot EDS analysis (Table 5), the following observations were noted. At spots 1 and 2 (Figure 16a), the material consisted primarily of copper (>99.70 wt.%) with trace amounts of aluminum (0.21 wt.%). Spots 3 and 4 were transitional regions, where Al and Cu content predominated, respectively. Measurement at spot 5, on the other hand, showed a clear predominance of aluminum atoms (99.21 wt.%) over a minimal amount of copper (0.79 wt.%). At spots 1 and 2 of the TCAP2 sample (Figure 16b), copper atoms predominated with a minimal amount of aluminum (<0.30 wt.%). Spots 3 and 4 were located in the transition region, which is a mixture of both metals with a predominance of copper elements (>65.00 wt.%). At spot 5, no Al atoms were detected (Table 5). At spot 1 of the TCAP3 sample (Figure 16c), copper atoms predominated (99.62 wt.%) with aluminum admixture (0.38 wt.%). Spots 2 and 3 were in the transition region, which is a mixture of both metals. Near the Al/Cu interfacial transition layer (spot 3), more copper elements (52.12 wt.%) were detected than at spot 2 (45.04 wt.%), where aluminum content predominated (54.96 wt.%). At spot 4, located near the Al/Cu interface, a predominant amount of copper (89.61 wt.%) was detected with a small presence of aluminum elements (10.39 wt.%). Spots 1 and 2 of the TCAP4 sample (Figure 16d) show a predominant copper content (>99.50 wt.%) with aluminum impurity (<0.50 wt.%). Spots 3 and 4 are characterized by comparable weight contents of Al and Cu. At spot 5, located in the EN AW-1050 layer, a predominant amount of aluminum (99.47 wt.%) with a copper impurity (0.53 wt.%) was found.

Summarizing the results of spot EDS analysis, no significant differences were observed between the TCAP processing routes. The only exception was sample TCAP2, which demonstrated a more deformed interface than the other samples as a result of two-pass TCAP. In the areas furthest from the Al/Cu interfacial transition layer, the presence of aluminum in the Cu-ETP plate material decreased, until it was completely absent from the Cu-ETP material layer. Based on the spot EDS analysis, it was confirmed that intermetallic compounds had formed at the Al/Cu layer interface. It is suspected that one of these compounds could be the η (AlCu) phase, one of the most common IMC formed during explosive welding of Al/Cu plates. Observations of the microstructure of explosively welded EN AW-1050/M1E plates revealed that processes occurring in the liquid state (intense mixing of molten metals) predominated over those observed in the solid solution [49]. Similar analyses of the chemical composition in the Al/Cu interfacial transition layer in EN AW-1050/M1E bimetalic plate revealed the occurrence of three crystalline equilibrium phases of the Θ (Al_2_Cu), η (AlCu), and γ (Al_4_Cu_9_) type [49].

#### 3.3.3. Microhardness of TCAP-Processed Samples

**Figure 17:**
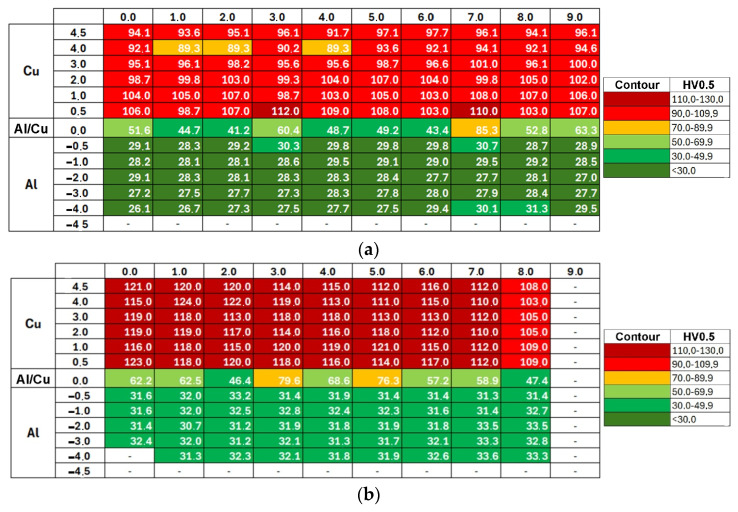

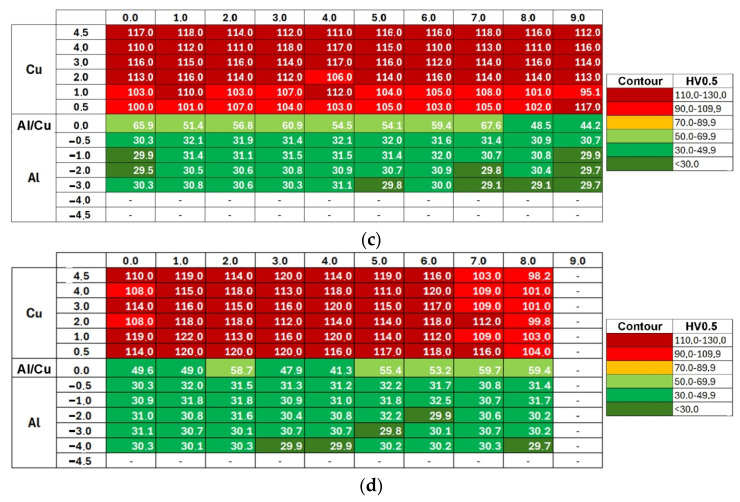
Vickers microhardness distribution map in the samples: (**a**) TCAP1, (**b**) TCAP2, (**c**) TCAP3, and (**d**) TCAP4.

Figure 17a shows the Vickers microhardness distribution map for the TCAP1 sample. Due to the severe deformation of the TCAP-processed sample, measurements could not be taken within 4.5 mm of the Al layer interface. The color scale allowed for a visual depiction of the effect of the TCAP process on the material microhardness. The microhardness in the Cu-ETP layer did not change significantly, but decreased in both the Al/Cu interface and the EN AW-1050 plate material. Microhardness measurements for the EN AW-1050 plate also showed a more uniform distribution across the entire plate thickness compared to the as-welded bimetallic plate, where microhardness for the EN AW-1050 plate material varied depending on the distance from the Al/Cu interface (Figure 9 and Figure 11). Analysis of the microhardness distribution map revealed the presence of individual values that outlied the general trend. 

**Figure 18:**
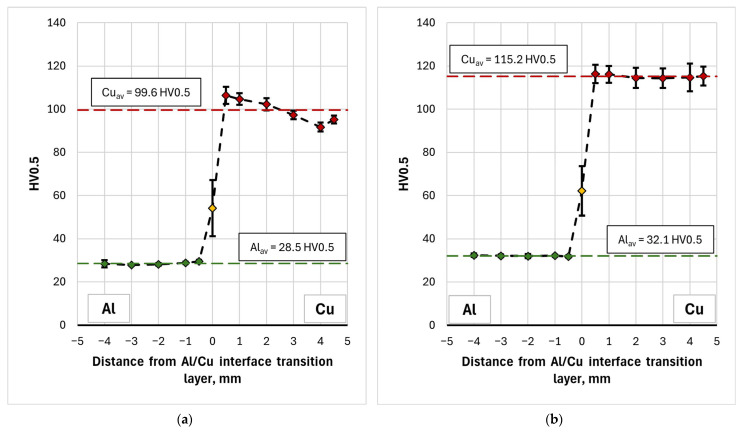

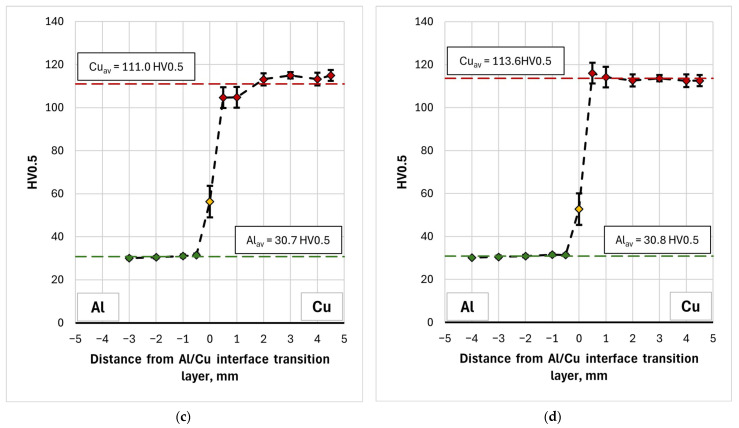
Distribution of average microhardness on the transverse section of samples: (**a**) TCAP1, (**b**) TCAP2, (**c**) TCAP3, and (**d**) TCAP4.

**Figure 19:**
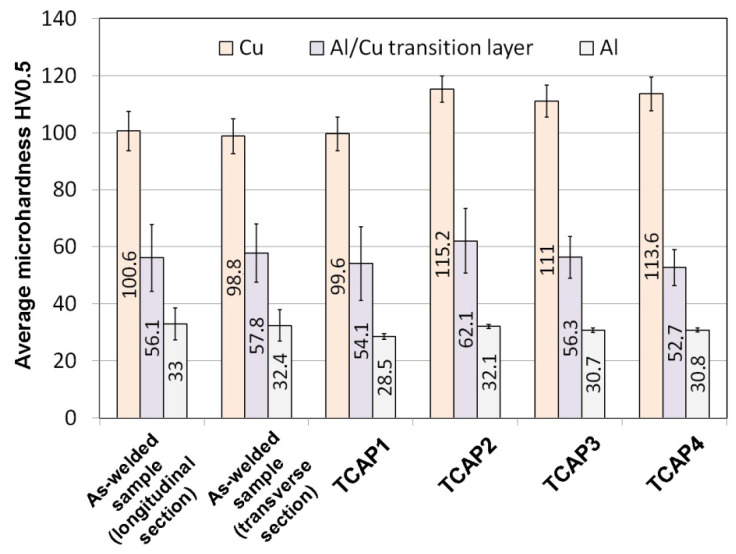
Comparison of the average microhardness of the tested materials.

Compared to the input material (as-welded), the aluminum layer after TCAP is characterized by a more uniform microhardness distribution, both in across the thickness and width of the sample (Figure 17). Furthermore, the microhardness increase in the area near the interface was insignificant compared to the aluminum layer in the as-welded material. A different observation was noted for the copper layer (Figure 17 and Figure 18). For samples TCAP2 and TCAP4, a uniform microhardness distribution was also observed in the tested part of the bimetallic material, particularly in the former. However, in the remaining cases, varying effects of TCAP process on the material’s microhardness were observed. For the TCAP1 sample, a similar pattern of microhardness decrease was observed with distance from the interface to the as-welded material. However, in the case of the TCAP3 sample, in which the Cu layer faced the die exit channel, microhardness increased with distance from the interface, particularly up to a distance of 3 mm. This observation indicates that the method of inserting bimetallic samples into the input channel in the TCAP process is a significant parameter influencing the microhardness of individual layers. Despite the use of a twisted output channel (Ψ = 30°), the purpose of which was to increase the total accumulated deformation in the material by imparting an additional torsional strain component to the billet [50,51], the applied output channel twist angle was too low, resulting in strain distribution in the material during extrusion. This contributed not only to the difference in the microhardness distribution across the copper layer thickness between samples TCAP1 (Al layer facing exit channel) and TCAP3 (Cu layer facing exit channel) processed in one pass, but also to its higher average value for the latter (Figure 19). Slightly better results and a more uniform distribution of microhardness over the Cu layer thickness was observed for the TCAP4 sample, where both layers faced the exit channel simultaneously (Figure 18d). 

Figure 19 compares the average microhardness values of TCAP-processed and as-welded samples. Using a single TCAP cycle did not result in a significant increase in the microhardness of the bimetallic material. Furthermore, a decrease in average microhardness was observed for both the Al layer and the Al/Cu interface, although the scatter of measured values for the interface, shown as whiskers on Figure 19 (standard deviation), is large enough to consider this difference insignificant. When examining the average microhardness of the Al layer in the as-received sample—excluding the region adjacent to the Al/Cu interface—it amounts to 29 HV0.5, which is identical to the value observed after the first TCAP cycle. This means that no significant strain hardening effect occurred in the aluminum layer. Moreover, in the area near the Al/Cu interface, microhardness of Al decreased (from 41 HV0.5 to 30 HV0.5). This can be attributed to the high stacking fault energy of aluminum, ranging from 150 to 230 mJ/m^2^ [52,53]. This indicates that aluminum has a high tendency to recover, which can occur during plastic deformation [54] especially since the material had already been subjected to significant deformation resulting from the explosive welding process (Al was a flyer plate). For the copper layer, which was located on the opposite side of the sample from the exit channel, the total material deformation effect was too low and it caused only a slight increase in microhardness. However, considering the SD values, depicted as standard deviation error bars in Figure 19, it can be concluded that there is no significant difference between the as-received and TCAP1 samples. A different effect was observed in the remaining samples, particularly in the TCAP2 sample subjected to two-pass TCAP (route Bc). An increase in average microhardness was observed for both the copper and aluminum layers, as well as the Al/Cu interface. This means that as a result of the second TCAP pass, significant strain hardening occurred in the entire sample. The average microhardness of the aluminum layer was lower than that of the as-received material. In the case of TCAP3 and TCAP4, the hardness of the aluminum layer, similarly to the TCAP1 sample, was lower than that of the as-received material. At the same time, the recovery effects in the aluminum layer were found to be lower, resulting in its higher average hardness (nearly 31 HV0.5). This can be due to the nature of the extrusion process with angular channels, which is characterized by the presence of strain distribution in the material throughout its thickness—even when applying additional torsional deformation, especially at the twist angles of helical part of the output channel equal to 10° [55]. This type of distribution, combined with different methods of sample insertion into the channel (sample orientation), may contribute to the occurrence of different phenomena and effects in the Al and Cu layers, depending on the sample orientation. Thus, it is possible that, in the case of the TCAP3 and TCAP4 samples, the energy delivered to the aluminum layer as a result of the TCAP process was lower, consequently limiting recovery-related effects in the Al layer. Neverthless, the statistical analysis indicated that the differences in microhardness values for Al layers were statistically significant and associated with a large practical effect (*p* < 0.001, Cohen’s d ≈ 0.82).

On the other hand, TCAP extrusion, especially with a larger number of passes (TCAP2) and appropriate positioning of the Cu layer relative to the output channel with one pass (TCAP3 and TCAP4), significantly affects its hardness, increasing it relative to the as-received material by about 15%. As shown in [56], plastic strain at levels 1.7 and 2.8, corresponding to the sum of the averaged ECAP and TE strain in billet [55] for one and two passes, respectively, contributes to a decrease in electrical conductivity relative to pure copper by only about 3–5%. Hence, the use of the TCAP method to increase the product hardness in the discussed range does not significantly contribute to a decrease in conductivity, especially if a single TCAP pass is used. Appropriate arrangement of Cu and Al layers relative to the exit channel allows for obtaining correspondingly higher hardness for copper, and therefore strength, after just one TCAP pass, only slightly lower than in case of TCAP2, with sufficiently high electrical conductivity.

A comparison of the results of mechanical property and microstructure investigations of explosively welded Al/Cu plates processed by ECAP [42] confirmed that TCAP enables the attainment of higher strain values. The TCAP process resulted in higher hardness values in all material layers compared to the sample deformed using the ECAP die. The microhardness of the aluminum layer during both TCAP and ECAP processing of Al/Cu plates was uniform across the thickness. However, in the TCAP process, depending on the orientation of the sample relative to the channel in the die, the microhardness values ranged from 28.5 to 32.1 HV0.5. In contrast, the microhardness of the Al plate subjected to ECAP ranged from 26.6 to 27.1 HV0.5. The microhardness of the ECAP-processed Cu plate ranged between 99.3 HV0.5 and 112.8 HV0.5 [55]. In the TCAP process, microhardness values between 99.6 HV0.5 (Figure 18a) and 115.2 HV0.5 (Figure 18b) were obtained. The morphology of the Al/Cu interfacial transition layer in samples processed via ECAP and TCAP was similar, with transition zones observed near the Al/Cu interface resulting from severe plastic deformation.

## 4. Conclusions

This article presents the results of research on the effect of SPD on the microstructure and mechanical properties (microhardness) of explosively welded Al/Cu plates. Various routes of the Twist Channel Angular Pressing process were considered. Based on the experimental results, key conclusions were reached as follows:

In the microstructure of as-welded samples, a wave-like profile of the weld interface and transition region with intermetallic phases were identified. No significant differences were observed in the morphology of the Al/Cu interfacial transition layer in transverse and longitudinal sections.

As a result of the explosive shock wave, the microhardness of the Cu-ETP and EN AW-1050 materials in the as-welded bimetallic plate were greatest near the Al/Cu interface and decreased toward the outer surfaces of both plates.

Microstructural analysis of TCAP-processed samples showed that the TCAP2 sample deformed along route Bc exhibited the most deformed the weld interface profile. The Al/Cu interfacial transition layer of TCAP2 sample was characterized by an irregular, highly distorted morphology, distinguishing it from the other samples tested. Although the bimetallic sample was subjected to severe shear strains, no cracking or delamination was observed in the Al/Cu interfacial transition layer.

The TCAP process caused mutual displacement of base metals in the vicinity of the weld interface. Quantitative spot EDS analysis showed no significant differences in the chemical composition of weld interfaces deformed with different routes.

The TCAP process resulted in a more uniform distribution of microhardness in the EN AW-1050 material compared to microhardness distribution in as-welded plate. The same conclusion can be made for the TCAP2 and TCAP4 samples, which confirms that for some TCAP routes, it is possible to ensure more uniform mechanical properties in both layers of the joined plates compared to the as-welded samples.

The orientation of the bimetallic material relative to the die exit channel and number of passes affected the final microhardness in the individual layers of explosively welded EN AW-1050/Cu-ETP bimetallic plate.

## Figures and Tables

**Table 1:** Chemical composition (wt.%) of EN AW-1050 aluminum plate [42].

Zn	Si	Mg	Fe	Ti	Cu	Mn	Others	Al
≤0.07	≤0.25	≤0.05	≤0.40	≤0.05	≤0.05	≤0.05	≤0.03	≥99.50

**Table 2:** Chemical composition (wt.%) of Cu-ETP copper plate [42].

O	Cu	Others	Bi	Pb
≤0.04	≥99.90	≤0.03	≤0.0005	≤0.005

**Table 3:** Designation of TCAP-processed samples.

Designation	Number of Passes	Sample Orientation at the Entrance to the Die Channel in the First Pass	TCAP Route
TCAP1	1	Al	-
TCAP2	2	Al	route B_C_
TCAP3	1	Cu	-
TCAP4	1	Al/Cu interface	-

**Table 4:** Results of the spot EDS analysis of chemical composition in the area of Al/Cu interfacial layer in transverse and longitudinal section of the explosively welded samples.

Section of Explosively Welded Sample	Spot No.	Chemical Composition, at.%	
		Al-K	Cu-K
Transverse section	1	1.38	98.62
	2	78.17	21.83
	3	99.26	0.74
	4	2.06	97.94
	5	27.05	72.95
	6	99.89	0.11
Longitudinal section	1	0.61	96.55
	2	98.88	0.64
	3	72.86	23.16
	4	22.81	69.46
	5	98.13	0.37

**Table 5:** Results of the spot EDS analysis of chemical composition in the area of the Al/Cu interfacial layer of TCAP-processed samples.

Sample	Spot No.	Chemical Composition, wt.%		Chemical Composition, at.%	
		Al-K	Cu-K	Al-K	Cu-K
TCAP1	1	0.21	99.79	0.49	99.51
	2	0.21	99.79	0.50	99.50
	3	61.26	38.74	78.83	21.17
	4	32.15	67.85	52.74	47.26
	5	99.21	0.79	99.66	0.34
TCAP2	1	0.24	99.76	0.56	99.44
	2	0.27	99.73	0.62	99.38
	3	29.96	70.04	50.19	49.81
	4	34.12	65.88	54.95	45.05
	5	100.00	0.00	100.00	0.00
TCAP3	1	0.38	99.62	0.90	99.10
	2	54.96	45.04	74.19	25.81
	3	47.88	52.12	68.39	31.61
	4	10.39	89.61	21.44	78.56
	5	99.26	0.74	99.69	0.31
TCAP4	1	0.28	99.72	0.65	99.35
	2	0.43	99.57	1.01	98.99
	3	52.60	47.40	72.32	27.68
	4	45.75	54.25	66.52	33.48
	5	99.47	0.53	99.78	0.22

## Data Availability

The original contributions presented in the study are included in the article, further inquiries can be directed to the corresponding author.
